# Examining the association of physical activity and mortality among recently hospitalized older adults with dementia

**DOI:** 10.1016/j.gerinurse.2024.06.024

**Published:** 2024-07-11

**Authors:** Brittany F. Drazich, Nayeon Kim, Merve Gurlu, Marie Boltz, Ashley Kuzmik, Elizabeth Galik, Barbara Resnick

**Affiliations:** a School of Nursing, University of Maryland Baltimore, USA; b School of Nursing, Penn State University, USA

**Keywords:** Physical activity, Dementia, Post-discharge, Mortality, Sedentary

## Abstract

**Introduction:**

This study aimed to examine the association between physical activity one month post discharge and mortality over the first-year post discharge among recently hospitalized older adults with dementia.

**Methods:**

For this descriptive sub-study, among 42 participants, we obtained physical activity data via accelerometry at one month post discharge and death status via phone call at 6 months and 1 year post discharge. We performed logistic regression.

**Results:**

We found that participants’ amount of time spent in physical activity one month post hospital discharge was not statistically significantly associated with mortality within the first-year post hospital discharge (OR=.996, CI=.992,1.000; p=.053). However, we did observe a strong trend.

**Conclusions:**

Given the small sample of participants, this trend is salient and should be examined in a larger sample. The results highlight a specific patient profile, recently hospitalized older adults with dementia, that would greatly benefit from physical activity interventions.

## Background

### Physical activity among people with dementia

Physical activity can prevent and treat at least 35 major chronic conditions such as heart failure, diabetes, and stroke,^1,2^ as well as preserve cognitive abilities.^3^ Physical activity is also a major predictor of the maintenance of physical function and the prevention of disability.^4,5^ Despite the clear association between physical activity and health, the majority of older adults do not meet the recommended physical activity guidelines.^6^ The physical activity of older adults with dementia is of particular concern given that they spend a disproportionate amount of time sedentary.^7^ A report by van Alphen and colleagues indicates that community dwelling older adults with dementia have 21.6% lower daily physical activity levels compared to their cognitively healthy peers.^8^

### Physical activity among recently hospitalized older adults with dementia

When older adults with dementia are hospitalized (approximately one in four hospitalized older adults has dementia^9,10^), they experience a multitude of barriers to physical activity such as exhaustion from their acute illness, bed alarms and other tethers, and disorientation due to an unfamiliar environment. Due to these barriers, older adults with dementia experience a loss of muscle mass and functional decline each day hospitalized, which can be difficult to restore following discharge.^11,12^ Thus, the period following hospitalization is an especially critical period for physical activity.^13,14^ However, it is estimated that recently hospitalized older adults with dementia spend 83.3% of their day sedentary.^15^ Given the low amount of physical activity among older adults with dementia post discharge,^15,16^ it is worthwhile to examine the association of their physical activity and health outcomes such as mortality, to inform post discharge recommendations and care. It is possible that physical activity following discharge is associated with less death through the prevention of morbidity (e.g., falls, infections).^1,2,17,18^

### Previous research and gap

Previous research is mixed related to the association of physical activity and mortality among older adults with dementia.^19–22^ Additionally, scarce research has specifically examined physical activity among older adults with dementia in the post discharge time period. This study aimed to fill the aforementioned research gaps through examining the association between physical activity post discharge and mortality among recently hospitalized older adults with dementia. We hypothesized that that greater amounts of physical activity at one month post discharge would be associated with lower mortality over the first-year post discharge.

## Methods

### Study design

This was a descriptive sub-study nested within the Function Focused Care for Acute Care Using the Evidence Integration Triangle (FFC–AC–EIT; ClinicalTrials.gov Identifier: NCT04235374) Study. The parent study, FFC–AC–EIT, is an intervention study which helps hospital nursing staff engage hospitalized patients with dementia in physical activity during care interactions. FFC–AC–EIT consists of four steps implemented over one year in the hospital setting: (1) environment and policy assessments of the hospital, (2) education of staff regarding the importance of physical activity for the patients with dementia, (3) establishing patient goals, and (4) mentoring and motivating staff, patients, and families. FFC–AC–EIT is described further by Resnick and colleagues (2022).^23^ For the sub-study, we initiated an additional in-person contact with participants one month after hospital discharge by measuring their physical activity using accelerometry. While this sub-study is nested in a hospital-based physical activity intervention study, we did not measure intervention effects, but rather the association of physical activity and mortality.

### Sample and setting

#### Parent study

In the FFC–AC–EIT parent study, hospitals were eligible to participate if they: (1) had at least one unit dedicated to general medical patients; (2) identified two registered nurses to be champions (one for day and one for evening shifts); (3) enabled staff to access email and websites via a phone, tablet, or computer; and (4) did not have a geriatric program (e.g., Acute Care for Elders, Hospital for Elder Life Program/ HELP) on the study units. Across two mid-Atlantic states, seven hospitals were included.

In the FFC–AC–EIT parent study, patients were eligible to participate if they were: (1) 65 years of age or older; (2) admitted to a medical unit for any medical diagnosis; and (3) screened positive for dementia based on: a score of ≤20 on the Saint Louis University Mental Status Examination (SLUMS),^24^ a score of ≥2 on the AD8 Dementia Screening,^25^ a score of 0.5 to 2.0 on the Clinical Dementia Rating Scale (CDR),^26^ and a score of 9 or greater on the Functional Activities Questionnaire.^27^ Patients were excluded from participation if they were: (1) enrolled in hospice; (2) were already on the unit for greater than 48 hours; (3) did not have a family member/care partner to contact; (4) anticipated having surgery; (5) had a major acute psychiatric disorder or significant neurological condition associated with cognition other than dementia; or (6) were COVID-19 positive. Patients with dementia could consent to the study if they passed the Evaluation to Sign Consent Measure, which assessed an individual’s capability to sign consent.^28^ If the patient with dementia did not pass the evaluation, we obtained the patient’s assent to contact their legally authorized representative who then participated in the consent process. Of note, during the consent process for the FFC–AC–EIT parent study, patients and/or their legally authorized representatives also consented to this sub-study.

#### Sub-study

For the sub-study, as we collected additional physical activity data on participants one month after hospital discharge, sub-study participants had additional inclusion and exclusion criteria. All parent study participants (both control and intervention group) were eligible for this sub-study if they were: (1) discharged from the hospital during the data collection period: October 1, 2021- April 1, 2022; (2) lived within 50 miles of the evaluator; and (3) reaffirmed their agreement to participate in this sub-study 30–60 days after hospital discharge via phone. Participants were excluded if they were: (1) in a hospital or a rehabilitation unit or (2) on hospice or died prior to the 30-day contact. During this data collection period, 133 parent study participants were eligible for this sub-study. Via phone, 42 participants and their care partners reaffirmed their agreement to participate and were included in this sub-study. Of the eligible parent study participants not included in this sub-study, 29 care partners and/or participants refused participation, 41 care partners did not return phone calls and could not be contacted, 15 participants were on hospice or died prior to the 30-day contact, and 6 participants were in a hospital or a rehabilitation unit.

### Procedures

Trained evaluators obtained data on the variable of physical activity through accelerometry. Between 30- and 60-days post hospitalization, an evaluator visited the participant at their home, assisted living facility, or nursing home, and applied the MotionWatch8 accelerometer to their non-dominant wrist. The evaluator then removed the MotionWatch8 after 5 days of wear and discussed with the care partner if any interruptions in wear time occurred. Trained evaluators obtained the variable of mortality status through reports from the participants’ care partners via phone calls at 6 months and 1 year post discharge. The parent study and sub-study were approved by the University of Maryland Baltimore Institutional Review Board.

### Measures

#### Physical activity

We obtained the predictor variable of “physical activity one month post discharge” through the first full 24 hours of accelerometry data collected by the MotionWatch8 when applied for 5 days between 30 and 60 days following hospital discharge. We decided to use the first *full* 24 hours of accelerometry data because some participants had instances of watch removal and reapplication throughout the 5 days. The MotionWatch8 is a lightweight activity monitoring device with a battery that lasts approximately one month. The MotionWatch8 contains a miniature accelerometer which measures counts of activity of the wrist providing a close correlation to whole body movement (1-minute epochs, 50 Hz).^29,30^ We grouped the counts of activity into time spent in sedentary, light, moderate, and vigorous activity, as determined by previously established set rates for older adults.^31^ Specifically, sedentary behavior is <178 counts per minute, light activity is 179–561 counts per minute, moderate activity is 562–1,019 counts per minute, and vigorous activity is >1,020 counts per minute. Of note, as participants spent scarce time in moderate and vigorous activity during this post-hospitalization period (see below), we grouped all physical activity (light, moderate, vigorous) together for analysis. Thus, we ended up with two variables from the MotionWatch8 data: 1) minutes spent in all physical activity and 2) minutes spent in sedentary behavior. Prior evidence supports the use of the Motionwatch8 based on test-retest reliability across 3 days and validity based on physical activity diaries.^29,30,32^

#### Mortality status

We obtained the outcome variable of “mortality status within a year post discharge” through care partner report of participant death or alive status at 6 months and 1 year post hospital discharge via phone. Mortality status was coded as 0 “alive” and 1 “death.” The care partners also reported cause of death.

#### Demographical variables

Via chart review, we measured comorbidities using the adapted Charlson Comorbidity Index, which is a summary of the total number of comorbidities. This Charlson Comorbidity Index ranged from 0–17, with higher scores indicative of more comorbidities.^33^ We measured cognitive impairment severity through patients’ completion of the Saint Louis University Mental Status Examination (SLUMS) at the time of parent study enrollment (during hospitalization).^24^ SLUMS scores range from 0–30, with a score of 0–19 or 0–20, depending on education, indicating dementia. For the group variable (control versus intervention group membership in the parent study) we coded the control group membership “0” and intervention group membership “1.”

### Data analysis

We first performed descriptive statistics including measures of central tendency, dispersion, and appropriate visualization approaches on each variable. We used a p-value significance level of ≤ .05 and used SPSS Version 26 to conduct all analyses.

To test the association between time spent in physical activity at one month post hospital discharge and death within the year post discharge, we initially performed a simple logistic regression. Next, we ran a multiple logistic regression, controlling for two variables highly associated with death, comorbidities (Charlson Comorbidity Index), and cognitive status (SLUMS),^34^ as well as group membership from the parent study (control versus intervention group).

## Results

### Description of sample

The sample consisted of 42 recently hospitalized older adults, 33 (78.6%) of whom identified as white and 23 (54.8%) of whom identified as male. The participants had an average age of 84.0 years (SD=7.9), an average of 1.7 (SD=1.0) comorbidities, and an average score of 7.0 (SD=5.9) on the SLUMS, which is indicative of severe cognitive impairment in this sample. See Table 1 for a more detailed description of the demographics of this sample.

One month following hospital discharge, within a specific 24-hour period, individuals with cognitive impairment spent an average of 16.6 minutes (SD=25.3) in vigorous activity, 59.5 minutes (SD=62.0) in moderate activity, 171.5 minutes (SD=107.2) in light activity, and 1,192.5 minutes (SD=180.1) in sedentary behavior. Dichotomized, the participants spent a total of 247.5 minutes (SD=180.1), or 17% of their day, in all activity and 1,192.5 minutes (SD=180.1), or 83% of their day, in sedentary behavior. A total of 17 of 42 (40%) participants died within the year following hospitalization. Of the 17 participants who died, 12 participants died within 6 months of hospital discharge and 5 participants died between 6 and 12 months post discharge.

### Association of physical activity one month post hospital discharge and mortality within the first year post hospital discharge

**Fig. 1.**
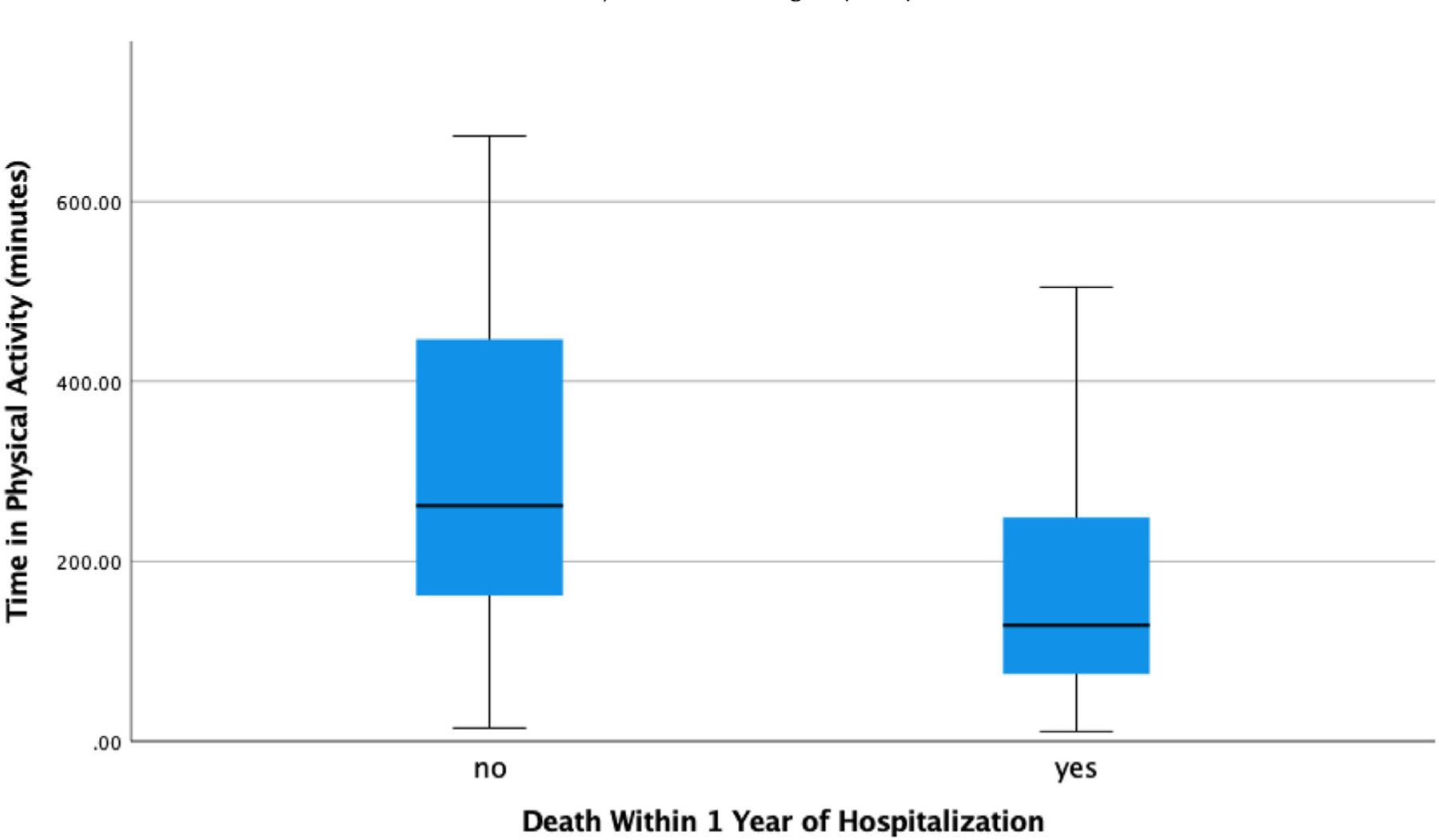
Association of time spent in physical activity and death.

**Fig. 2.**
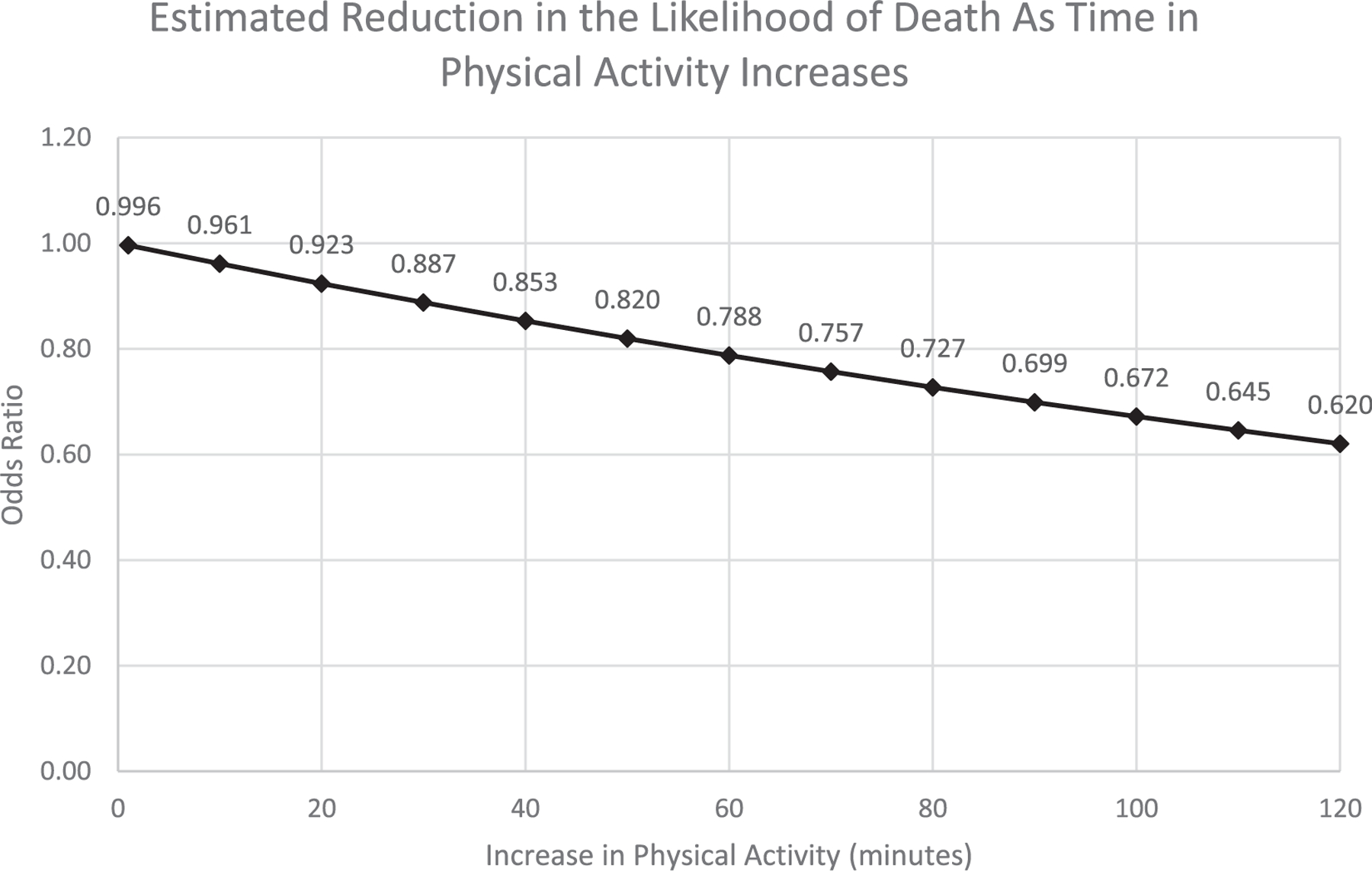
Estimated reduction in the likelihood of death as time in physical activity increases.

Through simple logistic regression, we found that participants’ amount of time spent in physical activity one month post hospital discharge was not statistically significantly associated with mortality within the first-year post hospital discharge (OR=.996, CI=.992,1.000; p=.053) (Table 2). However, we did observe a trend in which more physical activity one month post discharge was associated with lower mortality within the first-year post hospital discharge (See Fig. 1). In particular, the data suggests that as a recently discharged person with dementia increases their time spent in physical activity by 10 minutes, the odds that they will die over the next year decreases by 4% (See Fig. 2). For example, a particular participant who performed 15 minutes of physical activity per day one month after hospitalization had a 62% predicted probability of dying within the year. Alternatively, a particular participant who performed 673 minutes of physical activity per day one month after hospitalization had an 11% predicted probability of dying within the year. When testing the same association, physical activity one month post hospital discharge and mortality within the first-year post hospital discharge, and while controlling for variables that are associated with death (e.g., comorbidities and cognitive impairment severity) and parent study group membership (control versus intervention group), we again found no statistically significant association (OR=.997, CI=.992,1.001; p=.143). The causes of death for participants were advanced dementia or Alzheimer’s (n=4), infection (n=3), Parkinson’s Disease (n=2), fall (n=2), acute respiratory failure (n=1), diabetes (n=1), metastatic disease (n=1), kidney failure (n=1), congestive heart failure (n=1), and failure to thrive (n=1).

## Discussion

This study aimed to examine the association between physical activity one-month post discharge and mortality within a year post discharge among older adults with dementia. We obtained accelerometry data from older adults with dementia between 30- and 60-days post hospital discharge and spoke with their care partners by phone at 6 months and 1 year post discharge. We found that participants’ amount of time spent in physical activity one month post hospital discharge was not statistically significantly associated with mortality within the first-year post hospital discharge (OR=.996, CI=.992,1.000; p=.053). Although not statistically significant, we did observe a trend in which more physical activity one month post discharge was associated with lower mortality within the first-year post hospital discharge. Given the extremely small sample of participants, this trend is salient and should be examined in a larger sample. This trend complements previous literature which found that physical inactivity post discharge was associated with higher mortality among individuals with heart failure^35^ and COPD.^36^

We found that a total of 17 of 42 (40%) participants died within the year following hospitalization. Some of the 17 participants who passed away within a year of hospital discharge died from causes that possibly could have been prevented, at least in part, by physical activity. For example, physical activity has been associated with the prevention of infection,^37^ falls,^17,18^ respiratory failure,^38^ congestive heart failure,^39^ the management of complications associated with diabetes,^40^ and maintenance of cognitive ability.^3^ Nonetheless, death is a natural progression in life, and cannot be decelerated in some cases. In these cases, there is still value in engaging in physical activity to maintain a longer period of time at one’s highest possible level of cognitive and functional ability.^3,4^

This study’s major limitation is small sample size, which could limit the generalizability and replicability of the results. This small sample size also limited our ability to control for additional factors that might affect mortality such as delirium and discharge location. Additionally, as some members of our study sample received the Function Focused Care intervention in the hospital which may have influenced their post discharge behavior (parent study FFC-AC-EIT), researchers should use caution when drawing conclusions about the amount of physical activity performed by participants in this sample. Another limitation of this study was that we obtained information on the participants’ cause of death from their care partners, thus the causes of death reported in this study might vary from the official death certificate completed by the health care provider.

Despite these limitations, we were able to obtain objective data on the physical activity of recently hospitalized older adults with dementia for the purpose of examining the association between physical activity and mortality. Objectively examining physical activity following discharge in this population can be a particularly difficult endeavor given the difficulty in maintaining contact with people living with dementia as well as logistical issues in utilizing accelerometry for measurement once individuals are home. Even though we obtained data from a small sample and the results were not statistically significant, we observed a trend suggesting that physical activity at one month may be associated with death in the first-year post discharge.

Future research might focus on the physical activity timeframe, physical activity type, and physical activity “dose” that is needed to provide maximum post-hospitalization benefits. Additionally, researchers might also examine the association of post-hospitalization physical activity and quality-of-life days (e.g., increasing days without acute illness and/or disability). From a clinical perspective, the results from this study highlight a specific patient profile, recently hospitalized older adults with dementia, that would greatly benefit from being targeted for physical activity interventions.

## Conclusion

This study reported on the association of physical activity one month post hospital discharge and death within a year post discharge. While the association was not statistically significant, we did observe a trend in which more physical activity one month post discharge was associated with lower mortality in the first-year post discharge. These findings support the need for additional studies testing these associations longitudinally, and among larger and more diverse samples.

## Figures and Tables

**Table 1:** Demographics (N=42) participant characteristics.

Characteristics	Total N	Mean (SD)	n (%)
**Age**	42	84.0 (7.9)	
**SLUMS**	42	7.0 (5.9)	
**Base Comorbidities**	42	1.7 (1.0)	
**Baseline Barthel Index**	42	67.2 (30.6)	
**Gender**			
Men			23 (54.8%)
Women			19 (45.2%)
**Race/Ethnicity**			
White			33 (78.6%)
Black or African American			9 (21.4%)
**Death Within One Year, n (%)**			
No			25 (59.5%)
Yes			17 (40.5%)

Note. SLUMS = St. Louis University Mental Status Exam.

**Table 2:** Results of logistic regression of physical activity and death.

Association of Time Spent in Physical Activity One Month After Hospitalization and Death Within a Year, Bivariate Analysis, Nagelkerke R^2^ =.13, N=42				

*Predictors*	*Beta (SE)*	*Odds Ratio*	*95% CI*	*P Value*

All Physical Activity	−.004 (.002)	.996	.992, 1.000	.053
Association of Time Spent in Physical Activity One Month After Hospitalization and Death Within a Year, Controlling for Demographics, Nagelkerke R^2^ =.28, N=42				

*Predictors*	*Beta (SE)*	*Odds Ratio*	*95% CI*	*P Value*

All Physical Activity	−.003 (.002)	.997	.992,1.001	.143
Comorbidities	.631 (.376)	1.879	.899, 3.928	.093
Cognitive Impairment Severity	−.082 (.066)	.922	.809, 1.049	.218
Control* Vs. Intervention Group	−.977 (.920)	.376	.062, 2.286	.288

Note. CI=confidence interval; Reference Group “0” = Control Group
